# Characterization of Potential Adverse Outcome Pathways Related to Metabolic Outcomes and Exposure to Per- and Polyfluoroalkyl Substances Using Artificial Intelligence

**DOI:** 10.3390/toxics10080449

**Published:** 2022-08-04

**Authors:** Andreas-Marius Kaiser, Maryam Zare Jeddi, Maria Uhl, Florence Jornod, Mariana F. Fernandez, Karine Audouze

**Affiliations:** 1 Environment Agency Austria, 1090 Vienna, Austria; 2 National Institute for Public Health and Environment (RIVM), 3721 MA Bilthoven, The Netherlands; 3 Université Paris Cité, T3S, Inserm UMRS 1124, F-75006 Paris, France; 4 Centre for Biomedical Research, E-18016 Granada, Spain; 5 Department of Radiology and Physical Medicine, School of Medicine, University of Granada, E-18071 Granada, Spain; 6 Consortium for Biomedical Research in Epidemiology and Public Health (CIBER Epidemiología y Salud Pública, CIBERESP), Instituto de Salud Carlos III, E-28029 Madrid, Spain

**Keywords:** AOP-helpFinder, adverse outcome pathways, metabolic syndrome, per- and polyfluoroalkyl substances, AOP-wiki

## Abstract

Human exposure to per- and polyfluoroalkyl substances (PFAS) has been associated with numerous adverse health effects, depending on various factors such as the conditions of exposure (dose/concentration, duration, route of exposure, etc.) and characteristics associated with the exposed target (e.g., age, sex, ethnicity, health status, and genetic predisposition). The biological mechanisms by which PFAS might affect systems are largely unknown. To support the risk assessment process, AOP-helpFinder, a new artificial intelligence tool, was used to rapidly and systematically explore all available published information in the PubMed database. The aim was to identify existing associations between PFAS and metabolic health outcomes that may be relevant to support building adverse outcome pathways (AOPs). The collected information was manually organized to investigate linkages between PFAS exposures and metabolic health outcomes, including dyslipidemia, hypertension, insulin resistance, and obesity. Links between PFAS exposure and events from the existing metabolic-related AOPs were also retrieved. In conclusion, by analyzing dispersed information from the literature, we could identify some associations between PFAS exposure and components of existing AOPs. Additionally, we identified some linkages between PFAS exposure and metabolic outcomes for which only sparse information is available or which are not yet present in the AOP-wiki database that could be addressed in future research.

## 1. Introduction

The adverse outcome pathway (AOP) is a modern framework in toxicology and risk assessment [1] that was first described by Ankley and co-workers [2] and was later launched by the Organisation for Economic Co-operation and Development (OECD) in 2012 [3]. The conceptual idea of an AOP is to describe the existing knowledge of a molecular initiated event (MIE) that can be linked to an adverse outcome (AO) through multiple key events (KE) connected by key event relationships (KER) occurring successively at different biological levels [2]. AOPs are developed to support hazard assessment by organizing mechanistic knowledge regarding toxicological processes and making a better use of data derived from non-animal methods [4]. Today, multiple AOPs have been developed for different chemicals as stressors. All AOPs, independent of their status (from under development to validated by the OECD), are accessible on the AOP-wiki database (https://aopwiki.org/, accessed on 28 February 2022) [5].

Per- and polyfluoroalkyl substances (PFAS) are a very large group of xenobiotics that are used for a multitude of applications due to their unique physicochemical properties (e.g., heat and acid resistance, water and oil repellency, and high surface activity) (see Glüge and co-workers [6]). PFAS are persistent and ubiquitous in the environment, and measurable levels of some PFAS are detected in human populations globally [7]. Toxicological and epidemiological studies have reported that exposure to certain PFAS and/or their mixtures may potentially interfere with a wide range of adverse health effects [8]; for instance, thyroid disruption, reproductive toxicity, reduced birth weight, various metabolic outcomes, and decreased immune responsiveness [9,10]. While a number of studies reported that exposure to certain PFAS (e.g., PFOA, PFOS, PFHxS, and PFNA) may significantly result in dyslipidemia (i.e., elevated triglycerides and total cholesterol and increased low-density lipoproteins (LDL)), hypertension (i.e., elevated blood pressure), obesity (i.e., elevated waist circumference), and insulin resistance/hyperglycemia (i.e., elevated fasting glucose), some studies published conclusions with disparities [11,12]. These metabolic outcomes, also known as components of metabolic syndrome [12], contribute to the risk of developing cardiovascular disease (CVD), which is the leading cause of death worldwide [13,14,15]. In general, the underlying biological mechanisms that explain how exposure to PFAS leads to these reported adverse health outcomes remain inconclusive. Similar to other persistent organic pollutants (POPs), PFAS interact with various nuclear receptors, including peroxisome-proliferator-activated receptors (PPARs), estrogen receptors (ERs), and the pregnane X receptor (PXR); however, the main reported mechanism is PPAR activation [16,17]. Currently, only one AOP initiated by perfluorooctanoic acid (PFOA), described as stressor, is available and citable [18]. AOP-166 starts with the activation of PPAR alpha (PPARα) as an MIE linking it to the AO of pancreatic acinar tumors in rodents [18].

Within the Human Biomonitoring for Europe (HBM4EU) project (https://www.hbm4eu.eu, accessed on 8 June 2022), an innovative computational tool named AOP-helpFinder was created to help AOP construction [19]. The AOP-helpFinder tool is a hybrid approach based on text mining and graph theory [19,20,21]. This tool allows researchers to accelerate the identification of existing knowledge between an environmental stressor (e.g., a PFAS) and biological events involved in AOPs (i.e., MIE, KE, and AO) through the scientific published abstracts. Owing to the rapid increase in the amount of released chemicals in the environment, chemical pollution has been recognized as a growing peril and a potential risk to human health. With the increasing amount of scientific literature in the PubMed database, it becomes feasible and valuable to investigate in a systematic manner the published linkages between stressors and health outcomes. Combined with manual curation, the AOP-helpFinder also allows researchers to identify stressor–event relationships in full texts. In previous studies, the tool was applied to bisphenol compounds [19,20], to a set of pesticides [21], and more recently, to decipher non-validated methods to characterize endocrine-disrupting chemicals [22]. This tool, based on artificial intelligence, was recently updated in a collaboration between HBM4EU and OBERON (https://oberon-4eu.com/, accessed on 3 July 2022 [23]) and is now proposed as a web server (http://aop-helpfinder.u-paris-sciences.fr/index.php, accessed on 15 February 2022) [24].

In the present study, we applied the AOP-helpFinder to rapidly identify and collect abstracts published in peer-reviewed journals addressing associations between PFAS exposures and metabolic health outcomes. The aim of this work was to identify patterns and plausible links between PFAS and available events from the AOP-wiki as well as complementary in-house events (MIE, KE, and AO) from epidemiological, *in vitro*, and *in vivo* studies to support the development of future AOPs.

## 2. Materials and Methods

### 2.1. Application of the AOP-helpFinder

#### 2.1.1. Development of the Dictionaries of Stressors and Events to Be Screened

In order to explore the associations between events (e.g., “metabolic health outcomes”) and stressors of interest (i.e., PFAS), the AOP-helpFinder tool needs two input dictionaries that will be automatically screened in order to identify the co-occurrence in abstracts between at least one event and a stressor.

The stressor dictionary contained a list of 59 PFAS that was created by experts. The majority of the PFAS were selected in accordance with the HBM4EU Scoping Document [25], and the list was additionally complimented by the judgment of experts.

The second dictionary, including events related to metabolism, i.e., MIEs, KEs, and AOs, was subsequently generated. This dictionary contained two distinct lists of events: one using data from the AOP-wiki database and a second one using medical subject headings (MeSH) knowledge. To define the AOP-wiki event list, we went through the downloaded full list of events from the AOP-wiki database (as of March 2020) and selected 17 AOPs of interest linked to metabolism. These selected AOPs were mostly related to lipid metabolism and liver steatosis, which are related to metabolic outcomes. Moreover, one AOP for obesity and two AOPs for hypertension were added after a manual search. From this, a first list containing a selection of 154 events extracted from the AOP-wiki database (https://aopwiki.org, accessed on 27 October 2020) was created. To select these events, all event types (MIE, KE, and AO) were considered, and only the ones of interest (i.e., related to metabolic outcomes) were kept. The second list included 19 MeSH specifically related to metabolic syndrome.

#### 2.1.2. Automatic Screening of the Literature Using the AOP-helpFinder Tool—Multistep Procedure

First step: All available abstracts (>33 million) from the PubMed database were screened using the previously defined list of 59 PFAS in a .txt format (Table S1). Only abstracts mentioning at least one of the PFAS from the list were selected and extracted in xml format files using the PubMed API (https://pubmed.ncbi.nlm.nih.gov/, accessed on 27 October 2020). All the extracted .xml files were the ones that were used for the text-mining step, meaning identifying abstracts that co-mention at least one PFAS (from list one) and one biological event (from list two, Table S2) (see step two). Second step: The dictionary of events containing the two lists of biological terms related to metabolism was used to automatically screen the abstracts identified during the first step.

All runs (i.e., abstracts for each individual PFAS against both lists of events) were conducted using the default parameters (i.e., the screening of the full abstracts and the calculation of two scoring systems to keep the most relevant associations (see Carvaillo and co-workers [19])). Step three: Identified abstracts co-mentioning at least one PFAS and a biological event were further manually investigated. When necessary, the full publication was read to confirm the association between the chemical event and PFAS exposure presented in the abstract.

**Figure 1:**
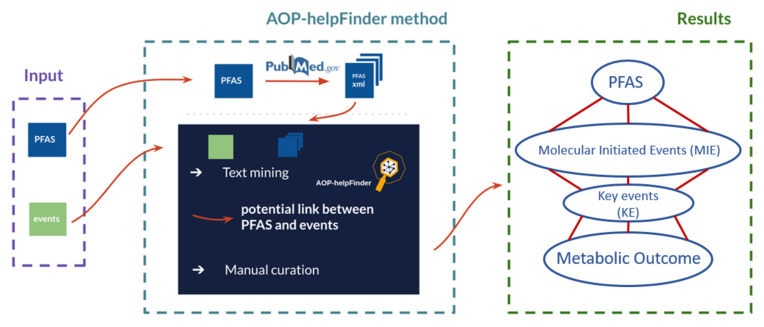
Workflow of the developed procedure to identify PFAS-mediated metabolic-related events. The proposed strategy is a multistep procedure: (1) Input: two lists (one with PFAS compounds and one with biological events (MIE, KE, and AO)) are needed as input data. (2) The AOP-helpFinder tool first extracts all available PubMed abstracts mentioning at least one PFAS from the first list. Then the tool retrieves biological events from the second list that are present in the selected abstracts and computes a distance score based on text mining and graph theory methods for prioritization. (3) As results, a linkage is proposed to help build AOPs induced by PFAS and related to metabolic outcomes.

The workflow of the developed procedure to identify PFAS-mediated metabolic-related events is shown in Figure 1.

## 3. Results

### 3.1. Linking PFAS to Adverse Effects

**Figure 2:**
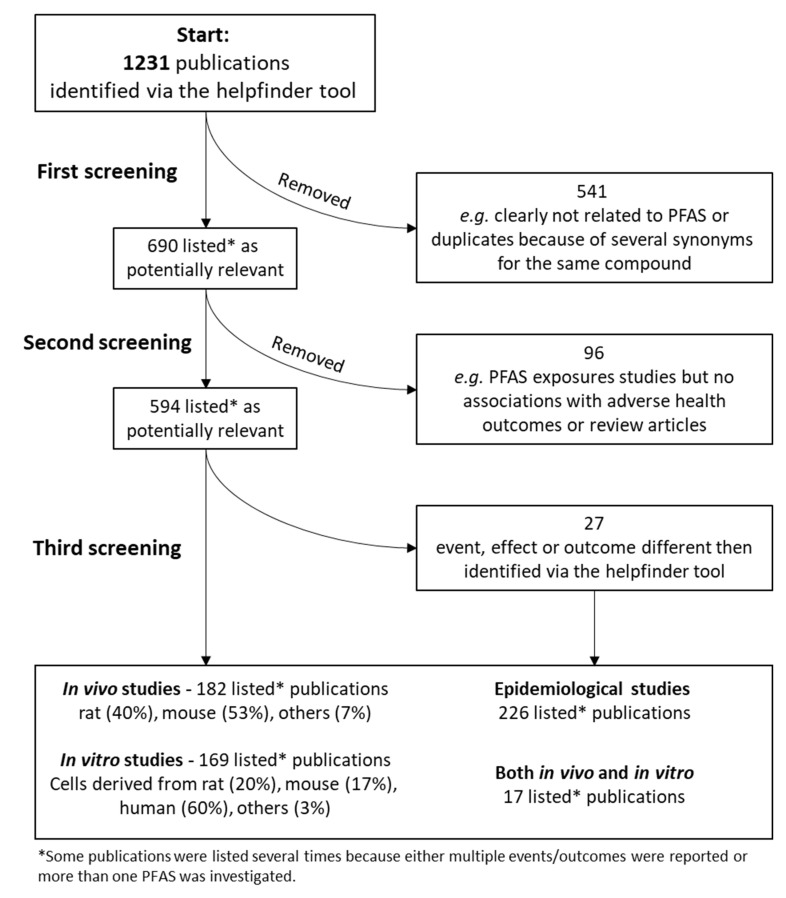
Flowchart of the collected publications and data using the AOP-helpFinder tool.

**Figure 3:**
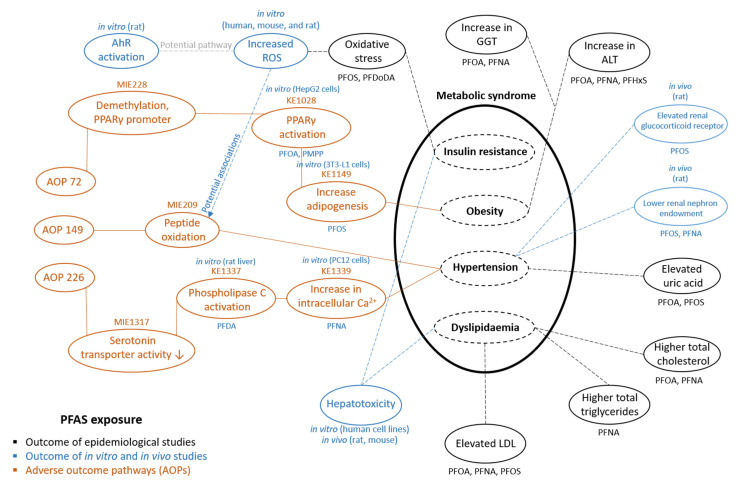
Identified connections between outcomes of epidemiological, in vivo, and in vitro studies on per- and polyfluoroalkyl substances (PFAS) and adverse outcome pathways (AOPs) from AOP-Wiki with a focus on the components of metabolic syndrome.

The application of the AOP-helpFinder resulted in more than 1200 rows of publications (see Figure 2), of which approximately one half were included for further investigations, and the other half were removed because they were either duplicates or not related to PFAS at all (e.g., PFAS were mentioned in the abstract but were not investigated in the study). Out of the 59 PFAS included in the stressor dictionary list, at least 27 PFAS (including 14 perfluoroalkyl acids (PFAAs, e.g., PFOA)) were addressed in the collected studies. The relatively small number of PFAS was characterized in 183 toxicological and 54 epidemiological studies. The most frequent health outcomes related to PFAS reported in the collected studies are summarized in Table 1 (*in vivo* studies), Table 2 (*in vitro* studies), and Table 3 (epidemiological studies) as well as in Figure 3.

#### 3.1.1. Collected Data from *In Vivo* Studies Using the AOP-helpFinder

As shown in Table 1, rats and mice were the most frequently studied animals in the *in vivo* toxicological studies, with hepatotoxicity (*n* = 29), PPARα activation (*n* = 19), decreased cholesterol (*n* = 8), and decreased body weight (*n* = 7) as the predominant studied outcomes. Toxicological reports concerning studies of other animals such as zebrafish were rather limited.

#### 3.1.2. Collected Data from *In Vitro* Studies Using the AOP-helpFinder

As for *in vitro* studies, human-based cell lines were most frequently used to investigate outcomes including increased levels of reactive oxygen species (ROS, i.e., oxidative stress; *n* = 12), hepatotoxicity (*n* = 11), and PPARα activation (*n* = 3)—see Table 2. In total, 10 studies used rat or mouse cell lines, and elevated ROS levels were detected after treatment with various PFAS. Furthermore, in rat cell lines, cell death and the activation of the aryl hydrocarbon receptor (AhR) were reported in some studies, and PPARα activation in mice was frequently observed.

#### 3.1.3. Collected Data from Epidemiological Studies Using the AOP-helpFinder

More than 15 epidemiological studies reported associations between PFAS exposures and hypertension and overweight/obesity and an inverse association with insulin resistance (see Table 3).

Most studies identified associations between PFOA (*n* = 9), PFOS (*n* = 5, including branched isomers), and PFNA (*n* = 4) and hypertension, while associations with other PFAS (e.g., PFBA, PFDA, PFHpS, PFDS, and MeFOSAA) and hypertension were less frequently reported. On the other hand, some studies did not identify associations between higher PFAS exposure levels (including PFOS (n = 4), PFOA (*n* = 3), PFHxS (*n* = 2), PFNA (*n* = 1), and EtFOSAA (*n* = 1)) and hypertension. However, elevated uric acids, as a biomarker of a higher risk of hypertension, were frequently reported in adults, adolescents, and children [26,27,28,29,30,31].

The reported associations between PFAS exposure and overweight/obesity were rather inconsistent and showed no clear trend. However, several indicators that can be linked to a higher risk of overweight/obesity were identified, e.g., increasing alanine aminotransferase (ALT) levels were found to be associated with higher PFOA, PFNA, PFHxS, and PFOS levels in adults, obese adults, and obese children (e.g., [30,32,33,34,35]). Elevated low-density lipoprotein (LDL), total cholesterol (TC), and triglycerides (TG) levels were also positively associated with several PFAS in adults and children (e.g., [36,37,38,39]), indicating higher risks of dyslipidemia.

Insulin resistance indicated via the homeostasis model assessment (HOMA) index was less frequently identified than inverse associations between higher PFAS exposure levels and insulin resistance. However, a few epidemiological studies also reported associations between a higher risk of gestational diabetes mellitus (GDM) and PFAS exposure. For instance, Sun and co-workers [40] found that higher PFOA and PFOS exposure levels were associated with a higher risk of type 2 diabetes mellitus (T2D). While Mancini et al. [41] found similar associations for PFOA and T2D, they did not find such associations for PFOS.

### 3.2. Linking PFAS to AOPs

We reduced the data complexity by creating a network diagram (see Figure 3) to simplify the identification of links between PFAS exposure and metabolic outcomes and related biomarkers.

Several effect biomarkers influenced by PFAS exposure were identified and were related with a value increasing the risk of metabolic disease, such as increased ALT levels, increased gamma-glutamyltransferase (GGT) levels, elevated uric acid levels, elevated TC, elevated TG, elevated LDL, elevated renal glucocorticoid receptor expression, elevated aryl hydrocarbon receptor (AhR) activation, increased reactive oxide species (ROS), or increase in intracellular calcium levels, in addition to decreased renal nephron endowment.

Comparing the identified molecular events and AOs to the three AOPs, including (1) AOP72: epigenetic modification of PPARG leading to adipogenesis, (2) AOP149: peptide oxidation leading to hypertension, and (3) AOP226: SSRI (selective serotonin reuptake inhibitor) leading to hypertension, which are related to either obesity or hypertension, showed that some of our identified events matched well to citable AOPs (Figure 3). For instance, the activation of PPARγ (KE1028 of AOP72) increased adipogenesis (KE1149 of AOP72), increased intracellular calcium (KE 1339 of AOP226), activated phospholipase C (PLC), or increased ROS, which are (potentially) related to peptide oxidation (MIE209 of AOP149).

We identified several studies that reported that certain PFAS, such as PFOS, PFOA, PFHxS, perfluoro-2-methyl-3-oxahexanoic acid (PMOH), and 3H-perfluoro-3-[(3-methoxypropoxy) propanoic acid] (PMPP), act as PPARγ agonists [42,43,44,45,46,47]. At least one study was identified reporting that PFOS exposure promotes adipogenesis [48], one reported that PFAS exposure increases intracellular calcium levels [49], one reported that PFDA exposure can activate PLC in rat livers [50] (KE1337 of AOP226), and various studies reported that PFAS exposure increases ROS levels [51].

## 4. Discussion

Considering the diversity of existing information on the toxicology of PFAS, we focused on exploring their relationships with metabolic outcomes from epidemiological studies as well as identifying molecular events and adverse outcomes via *in vivo* and *in vitro* studies using the AOP-helpFinder tool. Our findings indicated that PFAS exposure could negatively influence all components of metabolic syndrome, either individually or, more likely, simultaneously as a mixture.

Only three AOPs presented in AOP-Wiki, AO ID952 hypertension (AOP149), AO ID1343 increased hypertension (AOP226), and AO ID1447 obesity (AOP72), could be connected to various metabolic outcome markers (see Figure 3) that are also associated with PFAS exposure. As illustrated in Figure 3, the main identified events of this study are interconnected with each other, and our collected data show that some KE of existing AOPs can be initiated by PFAS as stressors as well. However, there were several AOPs provided in AOP-wiki that we could not include in this study because they are still under development and are therefore not citable. Our study outcome suggests that PFAS exposure directly influences biomarkers of dyslipidemia, hypertension, and obesity but not insulin resistance.

Although concentration differences in PFAS exposure, especially related to the region, sex, and age can vary significantly, we decided to reduce the complexity of the outcomes by omitting such subdivisions in general. For example, we found that studies that reported inverse associations between HOMA-IR and PFAS (mostly for girls) were conducted mainly in children [37,52] or adolescents [53]. On the other hand, studies that reported associations between elevated PFAS exposure levels and increased HOMA-IR were mostly conducted with adults or elderly individuals [54,55]. Since adults and elderly individuals are more prone to insulin resistance due to several age-related risk factors compared to children and adolescents, it is inconclusive to what extent these associations are related to the exposure to PFAS.

While it was not possible to identify some MIE/KE within the collected data using the AOP-helpFinder, probably due to the selected parameters, it was possible to fill some data gaps with some additional literature research using, e.g., search platforms such as Google Scholar. More recently, for example, one study reported that an exposure to a mixture of PFAS can lower the total brain dopamine concentration in mice as well as the tyrosine hydroxylase transcript level in mice [56], which are KE1450 and KE1452 from AOP72. Obesity, the AO of AOP72, is associated with PFAS exposure, as reported in some epidemiological studies. Tributyltin is the stressor of AOP72, and our collected data indicate that PFAS potentially influences the same molecular pathways, increasing the risk of obesity.

Furthermore, although we found that PFAS can increase the intracellular calcium levels (KE1339 of AOP226), it was shown that PFAS could cause a decrease in serotonin transporter activity (KE1320 of AOP226) [57]. While our study indicated some MIE/KE that are associated with PFAS exposure and metabolic outcomes, we also identified some gaps that could be addressed in the future, and we hope that our results can support the knowledge and developmental processes of AOPs. The results from *in vitro* and *in vivo* as well as epidemiological studies indicate that PFAS exposure is associated with hypertension, which is the AO of AOP149. However, to the best of our knowledge, it is still unknown if PFAS exposure decreases the expression of guanosine triphosphate cyclohydrolase 1 (GTPCH-1) or if it decreases tetrahydrobiopterin levels, KE935 and KE934 of AOP149. We also found that some events that are associated with PFAS exposure, e.g., an increase in alanine aminotransferase (ALT), are not included in any AOPs at present; further research could address this. Moreover, chronic inflammation (as assessed by different biomarkers such as proinflammatory adipokine, leptin, and/or proinflammatory cytokines IL-6 and IL-1β) has been described to play a crucial role in the development of metabolic disease. Very few studies have investigated whether PFAS exposure contributes to that process [58]. Novel clinical (adiponectin, leptin, and adiponectin-to-leptin ratio), molecular, and omic biomarkers have been inventoried within HBM4EU and proposed as biological indicators that could help to identify the role of PFAS in metabolic syndrome [59]. However, the evidence of their utility is still very limited [60]. For example, Fletcher and co-workers [61] showed associations between alterations in the levels of certain gene transcripts (e.g., NR1H2 (LXRB), ABCG1, and NPC1) involved in cholesterol metabolism and transport and PFOS/PFOA blood concentrations, suggesting consistency with the commonly observed elevations in blood total cholesterol related with PFAS exposure.

Metabolic perturbation outcomes, including lipid metabolism, glucose homeostasis, and thyroid hormone balance, might explain some of the observed associations between PFAS and birth outcomes [62,63]. In this regard, Kishi et al. (2015) found an inverse association between relatively low PFOS levels and lipid levels (TG and saturated (palmitic acid), monounsaturated (palmitoleic and oleic acids), omega 6 (linoleic and arachidonic acids), and omega 3 (α-linolenic acid and DHA) fatty acids) during pregnancy in a Japanese birth cohort [64].

Computational sciences, including systems toxicology, are of great interest to accelerate the identification of known relationships between the chemical exposome and biological events at various levels of biological organization. Today, a huge amount of data, e.g., derived from high-throughput and omics studies, are generated and available in databases and in the literature. The AOP-helpFinder tool, for which performance has been evaluated previously [19], has the ability to automatically and rapidly screen the scientific literature and allowed us to easily identify existing knowledge on PFAS and related metabolic events. Therefore, the development and assembly of AOPs is less laborious and time-consuming. These tools have also the potential to identify existing knowledge gaps. Nevertheless, AOP-helpFinder, similar to other computational approaches, is knowledge-based, and can only be used to identify existing, previously described, and available information. It is therefore important to mention that a lack of an association between a chemical and an event may be the result of less available data rather than the lack of effect. The AOP-helpFinder tool has some limitations, such as the text-mining part, which is performed only on the available abstracts of the published articles and will be optimized in the future with a version allowing the full texts of open data to be screened. Another limitation is the selection of the input data, which is crucial for the screening, as it is for all computational studies. For example, some protein acronyms could also be author names (e.g., AhR) or a disease name. Overall, utilizing the AOP-helpFinder tool allowed us to identify relevant publications and helped to realize a quicker and more relevant study compared to a fully manual study.

## 5. Conclusions

The strength of the study lies in the identification and selection of complex and generalized information data on PFAS related to metabolic outcomes in a time-efficient manner due to the AOP-helpFinder tool. We were able to show that when considering PFAS as stressors in the AOP framework the potential interrelationships of different events and pathways can be revealed. That seems relevant for the further development of AOPs and AOP networks and should be further explored. Furthermore, we managed to provide an overview of the available data in the published scientific literature, which will help scientists obtain an easier understanding of potential linkages between MIE, KE, and AO in the case of metabolic outcomes initiated by PFAS exposure. This overview shows where information is lacking and points out where further research would be beneficial to support AOP development and hazard assessment.

## Figures and Tables

**Table 1:** *In vivo* study outcomes related to metabolic syndrome.

Study Type (Number of Publ. Associated with Event)	Organism or Organism From Which Cells Were Originally Derived	Frequency (%)	Main Event Identified (Number of Publ. Associated with Event)	Substances Associated with Event
*In vivo* (114)	Rat	40	Hepatotoxicity (16)	PFCAs (C8-C12), PFOS
			Decreased cholesterol (8)	PFDA, PFOS
			PPARα activation (6)	PFOA, PFOS
	Mouse	53	Hepatotoxicity (13)	PFOA, PFOS, 6:2 FTSA, 6:2 FTCA, 6:2 Cl-PFESA, HFPO-TA, PFO2HxA, PFO3OA, PFO4DA
			PPARα activation (13)	PFCAs (C8-C10), PFHxS, PFOS
			Decreased body weight (7)	PFCAs (C8-C10), PFOS
	Others, e.g., hamster, guinea pig, monkey, zebrafish, carp	7	Hepatotoxicity, decreased body weight	PFOA, PFOS, PFDoDA

Abbreviations: PPAR = peroxisome-proliferator-activated receptors; PFCAs = perfluorocarboxylic acids; PFOA = perfluorooctanoic acid; PFDA = perfluorodecanoic acid; PFDoDA = perfluorododecanoic acid; PFHxS = perfluorohexane sulfonate; PFOS = perfluorooctane sulfonate; FTSA = fluorotelomer sulfonate; FTCA = fluorotelomer carboxylic acid; 6:2 Cl-PFESA = 6:2 chlorinated polyfluorinated ether sulfonate; HFPO-TA = hexafluoropropene oxide trimer; PFO2HxA = perfluoro-3,5-dioxahexanoic acid; PFO3OA = perfluoro-3,5,7-trioxaoctanoic acid; PFO4DA = perfluoro-3,5,7,9-butaoxadecanoic acid.

**Table 2:** *In vitro* study outcomes related to metabolic syndrome.

Study Type (Number of Publ. Associated with Event)	Organism or Organism From Which Cells Were Originally Derived	Frequency (%)	Main Event Identified (Number of Publ. Associated with Event)	Substances Associated with Event
*In vitro* (75)	Rat	20	Increased ROS (6)	PFOA, PFOS, 8:2 FTOH, PFOSA, PFDoDA
			Cell death (3)	8:2 FTOH, PFOS, PFOA, PFOSA
			Activation of AhR (1)	PFCAs (C8-C12), PFHxS, PFOS
	Mouse	17	PPARα activation (4)	PFCAs (C5-C9, C11-C12), PFHxS, PFOS
			Increased ROS (4)	PFOA, PFDA, PFOS
	Human	60	Increased ROS (12)	PFCAs (C8-C11), PFHxS, PFOS
			Hepatotoxicity (11)	PFOA, PFOS
			PPARα activity (3)	PFOA, PFOS, PFOSA
	Others, e.g., baikal seal, hamster	4	Increased ROS, PPARα activity	PFOA, PFOS, PFAAs (C4-C12)

Abbreviations: PPAR = peroxisome-proliferator-activated receptor; ROS = reactive oxygen species; AhR = aryl hydrocarbon receptor; PFCAs = perfluorocarboxylic acids; PFOA = perfluorooctanoic acid; PFDA = perfluorodecanoic acid; PFDoDA = perfluorododecanoic acid; PFHxS = perfluorohexane sulfonate; PFOS = perfluorooctane sulfonate; PFAAs = perfluoroalkyl acids; FTOH = fluorotelomer alcohol; PFOSA = perfluorooctane sulfonamide.

**Table 3:** Epidemiological study outcomes related to metabolic syndrome.

Event	Identified Publications via AOP-helpFinder		Identified Associations with PFAS (Number of Publ.)	No Associations Found (Number of Publ.)
Hypertension	Total number of publications:	20		
	Years:	2012–2020		
	Addressed hypertension or preeclampsia:	15	PFBA (1), PFOA (9), PFNA (4), PFDA (1), PFHxS (2), PFHpS (1), PFOS (3), br-PFOS (2), PFDS (1), MeFOSAA (1), total 12-PFAS (1),	PFOA (3), PFNA (1), PFHxS (2), PFOS (4), EtFOSAA (1)
	Did not address hypertension:	5	e.g., higher uric acid levels	
Overweight and obesity	Total number of publications:	15		
	Years:	1996–2019		
	Addressed overweight or obesity:	7	PFOA (3), PFNA (2), PFOS (1)	PFOA (1), PFNA (1), PFHxS (1), PFOS (2), PFOSA (1)
	Did not address overweight or obesity:	8	e.g., lower birth weight, elevated LDL or TC, study participants were obese in general	
Insulin resistance	Total number of publications:	11		
	Years:	2010–2019		
	Addressed insulin resistance:	11	↓ HOMA-IR↓: PFHxS (4), PFOS (1), PFOA (1), PFNA (2), PFDA (1), PFAA_4_ (1) ↑ HOMA-IR↑: PFHxS (1), PFOS (2), PFOA (1), PFDoDA (1) GDM: PFHxS (1), PFHpA (1), PFOA (1), PFNA (2), PFDoDA (1)	PFOA (2), PFNA (2), PFHxS (1), PFOS (2),
	Did not address insulin resistance:	0		
Type 2 diabetes mellitus	Total number of publications:	4		
	Years:	2018–2020		
	Addressed insulin resistance:	4	PFOS (1), PFOA (2)	PFOS (1)
	Did not address insulin resistance:			

Abbreviations: LDL = low-density lipoprotein; TC = total cholesterol; HOMA-IR = homeostatic model assessment for insulin resistance (↓= decrease, ↑= increase); GDM = gestational diabetes mellitus; PFBA = perfluorobutanoic acid; PFOA = perfluorooctanoic acid; PFNA = perfluorononanoic acid; PFDA = perfluorodecanoic acid; PFDoDA = perfluorododecanoic acid; PFHxS = perfluorohexane sulfonate; PFHpS = perfluoroheptane sulfonate; PFOS = perfluorooctane sulfonate; br-PFOS = branched perfluorooctane sulfonate; PFDS = perfluorodecane sulfonate; PFOSA = perfluorooctane sulfonamide; MeFOSAA = N-methyl-perfluorooctane sulfonamidoacetic acid; EtFOSAA = N-ethyl-perfluorooctane sulfonamidoacetic acid.

## Data Availability

Not applicable.

## Supplementary Materials

The following supporting information can be downloaded at: https://www.mdpi.com/article/10.3390/toxics10080449/s1, Table S1: list of Perfluoroalkyl and Polyfluoroalkyl Substances (PFAS) as stressors. Table S2: List of biological events.
