# Supplementary material for: Characterization of Potential Adverse Outcome Pathways Related to Metabolic Outcomes and Exposure to Per- and Polyfluoroalkyl Substances Using Artificial Intelligence
Source: Toxics. 2022 Aug 4;10(8):449. doi: 10.3390/toxics10080449 (PMC9412358; doi:10.3390/toxics10080449)
Supplement: Supplementary file 1 [file toxics-10-00449-s001.zip › toxics-1825571-supplementary.pdf]

Table S1: List of Perfluoroalkyl and Polyfluoroalkyl Substances (PFAS) as stressors

**Stressors**
Perfluoro-n-butanoic acid<br>Perfluoropentanoic acid<br>Perfluorohexanoic acid<br>Perfluoroheptanoic acid<br>perfluoro-heptanesulfonate<br>Perfluoropentanesulfonic acid<br>Perfluorooctanoic acid<br>Perfluorononanoic acid<br>Perfluoro-n-decanoic acid<br>Perfluorononanesulfonic acid<br>Perfluoro-n-undecanoic acid<br>Perfluorododecanoic acid<br>Perfluoro-1-butanesulfonate<br>perfluoro-1-decanesulfonate<br>Perfluoro-1-hexanesulfonate<br>Perfluorooctane sulfonic acid<br>perfluorooctane sulfonate<br>2-(N-Methyl-perfluorooctane sulfanamido) acetic acid<br>2-(N-ethyl-perfluorooctane sulfanamido) acetic acid<br>Perfluoro(2-ethoxy-2-fluoroethoxy)acetic acid ammonium salt<br>Perfluorotetradecanoic acid<br>perfluoro-n-tridecanoic acid<br>perfluoro-n-tetradecanoic acid<br>Krytox-H<br>Perfluorooctanesulfonamide<br>2-(Perfluorohexyl)ethyl acrylate<br>Polyfluoroalkyl phosphate mono-esters<br>Polyfluoroalkyl phosphate di-esters<br>Polyfluoroalkyl phosphate tri-esters<br>2-(Perfluoro-n-octyl)ethanol<br>2-(Perfluorodecyl)ethanol<br>2-(Perfluorooctyl)ethane-1-sulfonic acid<br>h,1h,2h,2h-Perfluorooctanesulfonic acid<br>Perfluoro-3,5,7,9-tetraoxadecanoic acid<br>Perfluoro-3,5,7,9,11-pentaoxadodecanoic acid<br>Ammonium 4,8-dioxa-3H-perfluorononanoate<br>Nafion Byproduct 2<br>6:2 chlorinated polyfluorinated ether sulfonic acid<br>Ammonium perfluoro-2-methyl-3-oxahexanoate<br>8:2 chlorinated polyfluorinated ether sulfonic acid<br>Trifluoroacetate<br>TFAA<br>C4-C4-PFPiA<br>Bis(nonafluorobutyl)phosphinic acid

C6-C8-PFPiA
Bis(perfluorohexyloctyl)phosphinic acid
C8-C8 PFPiA
Bis(perfluorooctyl)phosphinic acid
4-2 fluorotelomer sulfonic acid
3,3,4,4,5,5,6,6,6-Nonafluoro-1-hexanesulfonic acid
4-2 FTSA
Hexafluoropropylene Oxide Trimer Acid
HFPO-TA
Perfluorooctylphosphonic acid
2-(Perfluorohexyl)ethyl phosphonic acid
PFOPA
FL16.119
N-(2-methylcyclohexyl)-2,3,4,5,6-pentafluorobenzamide
PFECA
Perfluoro-1,2-propylene glycol and perfluoro-1,1-ethylene glycol, terminated with chlorohexafluoropropyloxy groups; Perfluoro(2-ethyloxy-ethoxy)acetic acid, ammonium salt
Perfluoro-2-methyl-3-oxahexanoic acid
PMOH
GenX
3H-perfluoro-3-(3-methoxypropoxy) propanoic acid
PMPP
ADONA
OBS
p-perfluorous nonenoxybenzenesulfonate
1-Octanol, 3,3,4,4,5,5,6,6,7,7,8,8,8-tridecafluoro-
6-2 FTOH
Perfluorotributylamine
FC43
Perfluoro-3,5,7-trioxaoctanoic acid
PFO3OA
1,1,2,2-tetrafluoro-2-(1,2,2,2-tetrafluoroethoxy)ethanesulfonic acid
NVHOS
4-defluoro-3-oxa-PFPeS
Hydro-EVE
polyfluoroalkyl ether carboxylic acid
2,2,3,3-tetrafluoro-3-((1,1,1,2,3,3-hexafluoro-3-(1,2,2,2-tetrafluoroethoxy)propan-2-yl)oxy)propanoic acid
Potassium 9-chlorohexadecafluoro-3-oxanonane-1-sulfonate
6-2 Cl-PFESA
Potassium 11-chloroeicosafluoro-3-oxaundecane-1-sulfonate
8:2 Cl-PFESA
Pentafluoropropionic acid
N-methylperfluoro-1 octanesulphonamide 1,1,2,2,3,3,4,4,5,5,6,6,7,7,8,8,8-Heptadecafluoro-N-methyl-1-octanesulfonamide (IUPAC)
n-MeFOSA
N-EtFOSA,
SULFLURAMID

N-EtFOSE<br>N-MeFOSE<br>PFODA<br>PfHxDA

Table S2: List of biological events

List of AOP-wiki events	List of HBM4EU events
stabilization PPAR alpha co-repressor<br>PPAR-gamma activation<br>Increased Reactive oxygen species<br>Increase FA Influx<br>Activation FAS<br>Activation Glucocorticoid Receptor<br>Up Regulation SREBF2<br>Up Regulation Unsaturated fatty acid<br>Down Regulation GSS gene<br>Down Regulation GST gene<br>Glutathione synthesis<br>Activation 3-hydroxy-3-methylglutaryl-CoA reductase gene<br>Perturbation of cholesterol<br>Glutathione homeostasis<br>Hepatotoxicity<br>Increase cytosolic fatty acid<br>Inhibit serotonin transporter activity<br>Decreased serotonin transporter activity<br>Decreased extracellular sodium<br>Decreased extracellular Na+<br>Decreased extracellular chloride<br>Decreased extracellular Cl-<br>Increased extracellular serotonin<br>Increased intracellular sodium<br>Increased intracellular Na+<br>Increased intracellular chloride<br>Increased intracellular Cl-<br>Decreased intracellular serotonin<br>Decreased packaged serotonin<br>Decreased synaptic release<br>Increased 5-HT3<br>Increased 5-hydroxytryptamine<br>Inactivated 5-HTR (serotonin receptors)<br>Reduce expression BDNF<br>Reduce expression Brain-derived neurotrophic factor<br>Decreased neuroplasticity	Insulin Resistance Syndrome<br>Syndrome X<br>Dysmetabolic Syndrome X<br>Metabolic Cardiovascular Syndrome<br>Type 2 Diabetes Mellitus<br>Insulin Sensitivity<br>Glucose Intolerances<br>Hyperglycemia<br>Insulin Resistance<br>Metabolic syndrome<br>Dyslipidemias<br>Dyslipoproteinemias<br>Hyperlipidemias<br>High Blood Pressure<br>Hypertension<br>Abdominal Obesity<br>Central Obesity<br>Liver Diseases<br>Thyroid Diseases

Increase cortisone<br>Reduced BDNF<br>Reduced Brain-derived neurotrophic factor<br>Activation 5-HT2A (Serotonin 2A)<br>Activate PLC<br>Activate Phospholipase C<br>Increase inositol triphosphate<br>Increase intracellular calcium<br>Activate calmodulin<br>Increase myosin light chain phosphorylation<br>Increase vascular smooth muscle contraction<br>Increase hypertension<br>Decreased extracellular serotonin<br>NFE2/Nrf2 repression<br>Increased steatosis<br>Reduced FXR activity<br>Reduced SHP activity<br>Activated LXR<br>Reduced PPARalpha<br>Reduced HSD17B4 activity<br>Reduced fatty acid beta oxidation<br>obesity<br>activation of CCAAT/enhancer-binding protein alpha<br>increased adipogenesis<br>decreased dopamine<br>decreased reward<br>decreased DNA methylation of tyrosine hydroxylase<br>decreased methylation of dopamine transporter promoter<br>chronic high fat diet<br>Steatohepatisis<br>Inhibition Fatty Acid Beta Oxidation<br>Increased Oncotic Necrosis<br>Tissue resident cell activation<br>Increased Pro-inflammatory mediators<br>Leukocyte recruitment<br>Leukocyte activation<br>Fatty Acid Beta Oxidation Decreased<br>Endocytotic lysosomal uptake<br>Activation LXR<br>Damaging Mitochondria<br>Mitochondrial dysfunction 1<br>Decreased Mitochondrial fatty acid beta-oxidation<br>Activation AhR	

Peptide Oxidation<br>Decreased PCK1 expression<br>Activation PPARα<br>peroxisome proliferator activated receptor promoter demethylation<br>Alkylation Protein<br>Activation PXR/SXR<br>Activation SCD-1<br>Activation SREBP-1c<br>Activation Stellate cells<br>Accumulation Triglyceride<br>Accumulation Fatty acid<br>Liver fibrosis<br>Liver Steatosis<br>Suppression VLDL secretion<br>Inhibition Mitochondrial fatty acid beta-oxidation<br>Increased Triglyceride formation<br>Accumulation Liver lipid<br>Suppression NR1I3<br>Activation SREBF1<br>Increased De Novo FA synthesis<br>Increased Liver Steatosis<br>Increased Liver Steatosis<br>Suppression HNF4alpha<br>Up Regulation SCD-1<br>Up Regulation FAS<br>Increased FA Influx<br>Up Regulation LDLR<br>Up Regulation low density lipoprotein receptor<br>Increased LDL uptake<br>Inhibition PPAR alpha<br>Up Regulation Acetyl-CoA carboxylase-1<br>Up Regulation ACC-1<br>Inhibition FoxA2<br>Down Regulation CPT1A<br>Down Regulation HMGCS2<br>Decreased Ketogenesis<br>Activation NRF2<br>Activation NR1H4<br>Activation SHP<br>Decreased DHB4/HSD17B4<br>Activation LXR alpha<br>Up Regulation CD36<br>Cell injury<br>Cell death<br>Decreased Cholesterol<br>Decreased De Novo Biosynthesis of	

Choleseterol<br>Decreased Uptake of Lipoproteins<br>Decreased Transport of Cholesterol to the Inner Mitochondrial Membrane<br>Decreased Testosterone Production by Adult Leydig Cells<br>Decreased sperm quantity and/or quality in the adult testis<br>Activation ChREBP<br>Accumulation Collagen<br>Up Regulation CYP1A1<br>Decreased PPARalpha transactivation of gene expression<br>Decreased Ketogenesis<br>Decreased production of ketone bodies<br>Not Increased Circulating Ketone Bodies<br>Increased Catabolism of Muscle Protein<br>Decreased Body Weight<br>Inhibition SREBP1c<br>Activation MTTP<br>Increased ApoB100<br>Increased Triglyceride<br>Disruption Lysosome<br>Synthesis De Novo FA<br>S-Glutathionylation eNOS<br>Uncoupling eNOS<br>Depletion Nitric Oxide<br>Decrease Tetrahydrobiopterin<br>Decrease GTPCH-1<br>Impaired Vasodilation<br>Increase Vascular Resistance<br>Hypertension<br>Decrease AKT/eNOS activity<br>Binding of antagonist PPAR alpha	
